# Spatial immune biomarkers in triple-negative breast cancer: emerging evidence, technology trade-offs, and barriers to clinical translation

**DOI:** 10.3389/fimmu.2026.1899098

**Published:** 2026-09-01

**Authors:** Liuhai Zeng, Wei Lin, Shihang Xue, Minzhi Chen, Haiyan Wu

**Affiliations:** Department of Breast and Thyroid Surgery, The Affiliated Xiangshan Hospital of Wenzhou Medical University, Ningbo, Zhejiang, China

**Keywords:** digital pathology, immune niches, immunotherapy, spatial biomarkers, tertiary lymphoid structures, triple-negative breast cancer, tumor-infiltrating lymphocytes

## Abstract

Triple-negative breast cancer (TNBC) is an immunogenic but clinically heterogeneous breast cancer subtype. Stromal tumor-infiltrating lymphocytes (TILs), PD-L1 expression, and tumor mutational burden provide useful but incomplete summaries of the tumor immune microenvironment. Spatial immune biomarkers retain tissue architecture and ask where immune cells are located, which cells they contact, and whether local neighborhoods are inflamed, excluded, suppressive, or organized into tertiary lymphoid structures (TLS). In this critical review, we examine CD8+ T-cell proximity, stromal versus intratumoral localization, suppressive myeloid and regulatory T-cell (Treg)-rich niches, TLS maturity, and spatial checkpoint co-expression in TNBC. We also compare multiplex immunofluorescence, multiplexed ion beam imaging by time-of-flight (MIBI-TOF), imaging mass cytometry, spatial transcriptomics, and hematoxylin-and-eosin (H&E)-based artificial intelligence in terms of spatial resolution, molecular depth, formalin-fixed, paraffin-embedded (FFPE) compatibility, scalability, reproducibility, and clinical feasibility. Most reported associations with prognosis or treatment response remain retrospective or exploratory, and their definitions are sensitive to sampling, segmentation, and threshold selection. The most clinically interpretable candidates are compartment-aware TIL measures, CD8+ T-cell-tumor proximity, and TLS-related features, but none is ready to guide treatment independently. Progress will require locked biomarker definitions, multisite analytical validation, external cohorts, and prospective trials with pre-specified spatial endpoints.

## Graphical Abstract

**Figure:**
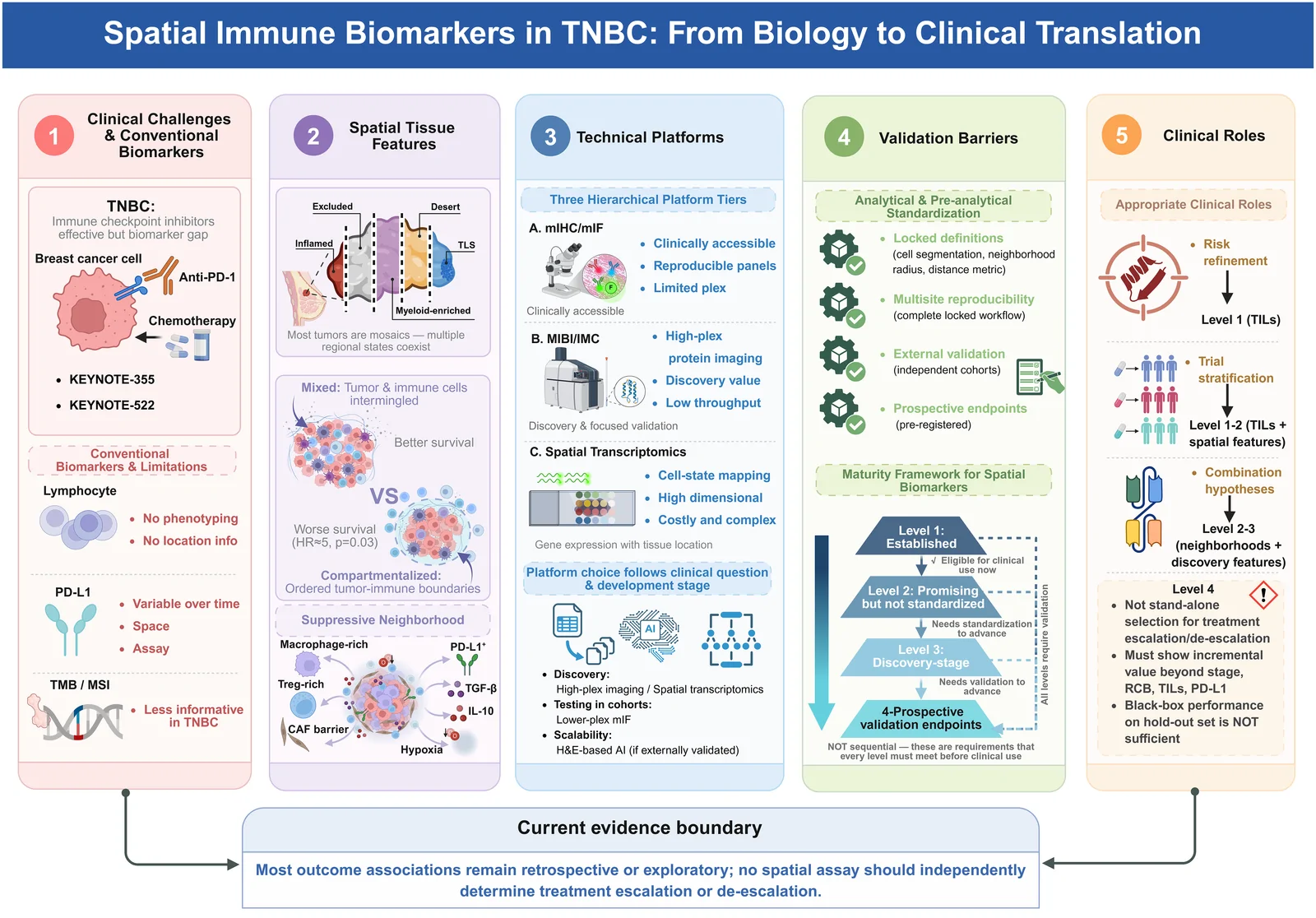
Spatial immune biomarkers in triple-negative breast cancer (TNBC), from biological organization to clinical translation. The evidence-maturity framework contains three levels: Level 1 (established contextual measures), Level 2 (promising but not standardized candidates), and Level 3 (discovery-stage signatures). Validation and implementation barriers are shown as overarching constraints that apply across all three levels, not as a fourth level. The figure also summarizes the limitations of conventional biomarkers, major spatial tissue patterns, profiling platforms, validation requirements, and appropriate clinical applications. Current evidence primarily supports risk refinement, clinical-trial stratification, and hypothesis generation rather than stand-alone treatment selection or escalation.

## 1 Introduction

Triple-negative breast cancer (TNBC) is defined by the absence of estrogen receptor (ER) and progesterone receptor (PR) expression and the lack of HER2 overexpression or amplification. Its genomic instability and frequent immune infiltration provided a strong rationale for early testing of immune checkpoint inhibitors (ICIs) (1). In advanced TNBC with a PD-L1 composite positive score (CPS) of at least 10, KEYNOTE-355 showed that pembrolizumab plus chemotherapy prolonged median overall survival from 16.1 to 23.0 months (2, 3). The atezolizumab experience was more complex. Atezolizumab plus nab-paclitaxel received accelerated US approval in 2019 on the basis of IMpassion130 (4, 5). The negative IMpassion131 trial challenged the generalizability of that benefit (6). The sponsor subsequently withdrew the US TNBC indication after discussions with the FDA (7, 8). In early-stage TNBC, KEYNOTE-522 improved pathological complete response and event-free survival (9, 10); at 60 months, estimated overall survival was 86.6% with pembrolizumab-chemotherapy and 81.7% with placebo-chemotherapy, with P = 0.002 (11). These trials establish the clinical importance of antitumor immunity while also illustrating the limitations of current predictive biomarkers.

These clinical trials also exposed a biomarker gap. Patients with the same PD-L1 category or similar stromal TIL percentages can experience markedly different outcomes. Immune abundance alone does not indicate whether cytotoxic cells can enter tumor nests, engage malignant cells, and sustain effector function. Lymphocytes confined to peripheral stroma, trapped behind cancer-associated fibroblast (CAF) and extracellular-matrix barriers, or concentrated around suppressive macrophages are biologically different from CD8+ T cells in direct contact with tumor cells. Likewise, PD-L1 positivity can mark an interferon-activated immune interface, an inhibitory myeloid compartment, or both. A single percentage score cannot distinguish these spatial contexts.

Spatial immune biomarkers should therefore be considered extensions of established immunopathological assessments rather than replacements. Their potential value lies in preserving tissue structure while quantifying cell state, compartment, distance, and multicellular organization. The clinically relevant question is not simply whether immune cells are present, but whether effective cells are positioned to recognize and eliminate tumor cells and whether that configuration persists during treatment (12, 13).

To make this evaluation explicit, we organize the evidence around a three-level maturity framework: Level 1 comprises clinically established contextual measures; Level 2 comprises biologically promising candidates that are not yet standardized; and Level 3 comprises discovery-stage signatures (**Table 1**). Validation and implementation barriers are not a fourth maturity level. They are overarching constraints that apply across Levels 1–3 and determine whether a candidate can progress from biological plausibility to a validated clinical test. The review therefore considers established biomarkers and the case for spatial extension, the technologies used to measure spatial organization, promising and discovery-stage candidates, evidence linking spatial features to treatment outcome, and the cross-cutting barriers that remain before clinical use.

**Table 1:** Candidate spatial immune biomarkers in TNBC across the three-level evidence-maturity framework and principal limitations.

Biomarker	Spatial definition	Method	Potential use	Evidence maturity	Main limitation
Stromal TILs and compartment-aware extensions	Stromal, invasive-margin, and intratumoral lymphocyte distribution	H&E; mIHC/mIF	Standardized prognosis/response assessment with exploratory spatial refinement	Level 1 for standardized stromal TIL scoring; compartment-aware extensions remain Level 2 (16, 17, 22, 29)	Compartment annotation and sampling vary
CD8+ T-cell-tumor proximity	Continuous distribution of distances or contact frequency	mIF; MIBI/IMC; AI	Measure executable cytotoxic access	Level 2 - Promising; retrospective/exploratory (34, 35)	Segmentation, distance metric, and cutoffs are not standardized
Immune exclusion	Immune cells outside tumor nests or behind stromal/myeloid barriers	H&E; multiplex imaging; AI	Identify trafficking or barrier-dominant resistance	Level 2 - Promising; definitions heterogeneous (12, 24)	Tumor-stroma boundaries and regional coexistence complicate classification
Spatial checkpoint state	Checkpoint expression by cell type and at tumor-immune interfaces	IHC; mIF; IMC; spatial transcriptomics	Distinguish adaptive activation from dysfunctional or suppressive states	Level 2 for limited-panel spatial states; high-dimensional multi-checkpoint signatures remain Level 3 (12, 23–26)	Assays, cell attribution, co-expression rules, and sampling differ
Treg/myeloid suppressive niche	FOXP3+ Tregs, tumor-associated macrophages (TAMs), or myeloid-derived suppressor cell (MDSC)-like cells near CD8+ cells or tumor	mIF; IMC; spatial transcriptomics	Generate hypotheses for myeloid- or stroma-directed combinations (31, 32)	Levels 2-3 - Discovery to promising (31, 32)	Marker-defined phenotypes are plastic and do not prove suppressive function
TLS-associated niche	Organized B-cell, T-cell, and dendritic-cell structures with maturity and location	H&E; IHC/mIF; spatial transcriptomics	Prognostic refinement and possible ICI-response enrichment	Level 2 - Promising; not standardized (42, 43, 52)	TLS definition, maturity, location, and sampling require harmonization
Spatial immune-state program	Interferon, antigen-presentation, hypoxia, and checkpoint programs in situ	Spatial transcriptomics/proteomics	Link geography to functional state and treatment trajectory	Level 3 - Discovery-stage (51, 52)	Platform sensitivity, batch effects, targeted panels, and cost
AI-derived immune geography	Composite density, distance, border, and morphology features	Digital pathology on H&E or multiplex images	Scalable screening or trial stratification after validation	Levels 2-3 - Discovery to promising (55)	Explainability, domain shift, and external/prospective validation

## 2 Established (level 1) immune biomarkers and the need for spatial context

TILs remain the most mature immune biomarker in breast cancer (14, 15). The International Immuno-Oncology Biomarker Working Group standardized stromal TIL assessment on H&E sections, and subsequent recommendations extended the framework to residual disease and carcinoma *in situ* (16, 17). Higher stromal TIL levels are consistently associated with favorable prognosis in early TNBC (18, 19). This association is also evident in young, node-negative patients who did not receive systemic therapy (20). Higher TIL levels are associated with higher rates of pathological complete response after neoadjuvant chemotherapy (21, 22). TIL scoring is inexpensive and readily integrated into routine pathology. Its deliberate simplicity is also its principal limitation: standard scoring does not phenotype individual cells and provides limited information about their location within tumor nests, invasive margins, and stromal compartments. Two tumors can therefore receive the same stromal TIL score despite very different opportunities for tumor-immune contact.

PD-L1 presents a related but distinct problem. It is a clinically used companion biomarker in advanced or metastatic TNBC, yet its expression varies over time, between tissue compartments, and across assays. KEYNOTE-355 uses CPS, whereas IMpassion130 used immune-cell PD-L1 staining with the SP142 assay; these approaches are not interchangeable (2, 4). Moreover, PD-L1 is only one component of a broader inhibitory network. Additional molecules such as PD-1, TIM-3, and LAG-3 have been examined on breast-cancer TILs (23). Spatial studies show that checkpoint interpretation depends on cell type, co-expression pattern, activation state, and local architecture (24). Broader TNBC reviews and outcome-linked microlandscape studies extend this inhibitory landscape to CTLA-4, TIGIT, IDO, and other targets, reinforcing the need for cautious contextual interpretation (25, 26). Current evidence supports limited-panel spatial checkpoint states as promising but not standardized (Level 2), whereas high-dimensional multi-checkpoint signatures remain discovery-stage (Level 3); neither should be interpreted as proof that a specific checkpoint combination predicts response.

Tumor mutational burden, microsatellite instability, and mismatch-repair deficiency are less frequently informative in TNBC than in several other solid tumors (1, 25). These biomarkers estimate antigenic potential but do not reveal whether an effective immune response is organized within the tissue (27, 28). Spatial analysis can distinguish intratumoral from stromal immune cells, quantify cytotoxic-cell proximity to malignant cells, identify Treg- and macrophage-rich suppressive neighborhoods, and characterize TLS-associated antigen presentation and B-cell maturation (12, 26–28).

Sampling is a major biological and methodological constraint. TNBC frequently contains adjacent immune-inflamed, immune-excluded, suppressive, and immune-poor regions (13, 26). A core-needle biopsy can therefore overestimate or underestimate the whole-tumor landscape, and pretreatment tissue may differ substantially from residual disease after therapy (13, 26). This becomes clinically consequential when a biomarker is proposed for treatment escalation or de-escalation. Spatial readouts should demonstrate reproducible, additive information beyond TIL and PD-L1 rather than functioning as costly surrogates for existing scores (15, 29).

## 3 Spatial organization of TILs and immune niches in TNBC: promising, not yet standardized candidates (level 2)

**Figure 1:**
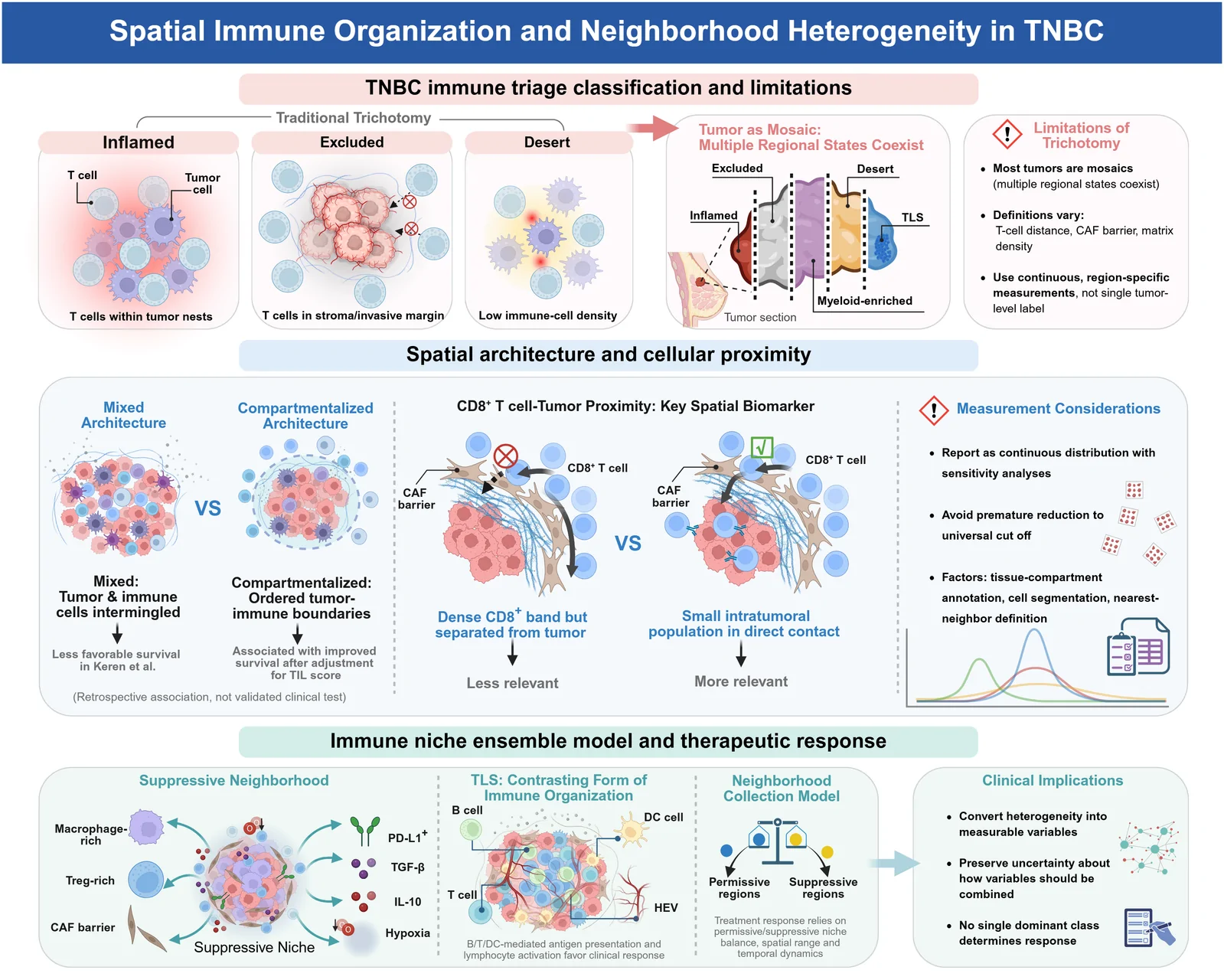
Spatial immune organization in TNBC: regional phenotypes, immune neighborhoods, and sources of computational variability.

The inflamed, excluded, and desert framework is useful for orientation but should not be treated as a fixed biological taxonomy. Most tumors are mosaics in which several regional states coexist. Definitions also vary across studies: immune exclusion has been operationalized using T-cell distance from the tumor boundary, CAF- or matrix-dense stromal barriers, or the absence of intratumoral CD8+ cells. Inflamed regions generally contain lymphocytes within tumor nests or at active tumor-immune interfaces; excluded regions contain immune cells that remain in stroma, at invasive margins, in perivascular niches, or behind cellular and extracellular barriers; desert regions have low immune-cell density. These states are better represented by continuous and region-specific measurements than by a single tumor-level label (30) (**Figure 1**).

Keren and colleagues provided an influential demonstration using MIBI-TOF to quantify 36 proteins in tumors from 41 patients with TNBC (12). Tumors with similar total immune infiltration showed either mixed architectures, in which tumor and immune cells were intermingled, or compartmentalized architectures with ordered tumor-immune boundaries. Compartmentalized organization was associated with longer survival (Cox regression hazard ratio = 4.97 for mixed versus compartmentalized tumors; P = 0.03) and provided information independent of pathological TIL scoring, although the small retrospective cohort limits the precision and generalizability of this estimate. Subsequent imaging mass cytometry and multiplexed pathology studies confirmed that phenotype, regional organization, and cell-cell relationships contain information beyond bulk abundance (26, 31). Multiplatform studies reinforced this principle while also exposing assay-dependent variation (32, 33). These findings support biological plausibility; they do not yet establish a clinically validated spatial test.

CD8+ T-cell-tumor proximity is one of the most interpretable promising, not-yet-standardized candidate spatial biomarkers. Cytotoxic lymphocytes generally require physical access to malignant cells, so a dense CD8+ band separated from the nearest tumor nest may be less relevant than a smaller intratumoral population in direct contact with tumor cells. Recent breast cancer studies have associated quantitative proximity measures with neoadjuvant response (34, 35). However, reported distances depend on tissue-compartment annotation, cell segmentation, nearest-neighbor definitions, and the selected threshold. Proximity should therefore be reported as a continuous distribution with sensitivity analyses rather than reduced prematurely to a universal cutoff.

At present, no single proximity statistic or universal cutoff has consensus status. Emerging SITC image-analysis and STORMI reporting recommendations instead emphasize locked end-to-end workflows, documented tissue and cell segmentation, phenotype rules, region-of-interest selection, pixel-to-micron calibration, quality control, and shareable analysis parameters (36–38). For harmonization, laboratories should pre-specify whether distance is centroid-to-centroid or edge-to-edge, the tumor compartment and eligible cell populations, and the observation window. The empirical cumulative distance distribution or a multi-radius contact curve can then be summarized by a small set of pre-specified quantities, such as median and lower-quartile distance, area under the curve, or the proportion of CD8+ cells within specified radii, with edge correction and density-adjusted point-process analyses when the question concerns enrichment beyond chance. Cross-laboratory agreement should be quantified using variance-component or mixed-effects models, intraclass correlation coefficients, repeatability coefficients, and Bland-Altman limits, followed by sensitivity analyses that perturb segmentation and radius choices. This approach preserves continuous information while allowing a later clinically validated cutoff to be locked.

Suppressive neighborhoods, a candidate spanning Levels 2-3, add further complexity. Treg- and macrophage-rich regions can coexist with high TIL levels and PD-L1 positivity, while TGF-beta, IL-10, dysfunctional antigen presentation, stromal barriers, and hypoxia limit cytotoxic activity (39, 40). TLS (promising, not standardized) represent a contrasting form of immune organization in which B cells, T cells, and dendritic cells support local antigen presentation and lymphocyte activation (41). TLS presence and maturity have been associated with favorable outcomes in breast cancer (42, 43), but definitions based on morphology, marker panels, maturation stage, and intratumoral versus peritumoral location remain inconsistent (23, 44).

A more faithful model treats each tumor as a collection of immune neighborhoods. One region may contain malignant cells, dendritic cells, and cytotoxic lymphocytes consistent with local recognition; another region in the same specimen may be dominated by macrophages, Tregs, activated fibroblasts, or hypoxia. Treatment response may depend on the balance, spatial extent, and temporal evolution of permissive and suppressive regions rather than on a single dominant class. This neighborhood view converts heterogeneity into measurable variables while preserving uncertainty about how those variables should be combined.

To translate a neighborhood map into trial eligibility, discovery should be followed by deliberate dimensionality reduction rather than selection of a single dominant neighborhood. A practical patient-level score could combine a small, pre-specified set of features: the area-weighted proportions of permissive and suppressive neighborhoods, a CD8+-tumor contact or distance summary, mature TLS density, and a heterogeneity term such as between-region variance or worst-quartile suppressive burden. Features should be aggregated across anatomically sampled regions with weights based on evaluable tissue area, standardized using parameters fixed in a training cohort, and combined using a locked penalized-regression or point-based model. The continuous score can then be calibrated to predicted response and converted to low, intermediate, and high strata, or to a binary eligibility rule, using cut points selected before external validation. A tissue-adequacy rule and explicit no-call category are essential when the sampled area is insufficient; sampling uncertainty should be carried into the score rather than hidden by averaging.

## 4 Technologies for spatial immune biomarker discovery: tools used across levels 1-3

Multiplex immunohistochemistry and multiplex immunofluorescence (mIHC/mIF) are among the most clinically accessible spatial platforms because they are compatible with routine FFPE tissue, use familiar antibody-based workflows, and can quantify phenotypes and distances at single-cell resolution (45). Conventional panels usually contain approximately 4–8 markers, although larger optimized panels are possible. The six-site MITRE study demonstrated that a locked six-plex assay can achieve intersite concordance for breast-cancer cell-density measures of approximately R² = 0.75 and for PD-1/PD-L1 proximity of R² = 0.82 (46). These results show that reproducibility is achievable under controlled conditions, but panel-specific antibody validation, autofluorescence, staining order, scanner calibration, thresholding, and image-analysis pipelines remain important sources of variation.

MIBI-TOF and imaging mass cytometry (IMC) use metal-isotope-labeled antibodies to profile substantially more proteins than conventional fluorescence panels. MIBI-TOF has been used for 36-plex TNBC imaging (12), whereas contemporary IMC studies commonly quantify 30–50 proteins (13, 32). This capacity builds on the original subcellular-resolution IMC framework (47). Standard IMC acquires tissue at approximately 1 μm resolution; recently described high-resolution IMC can reach below 350 nm but requires oversampling and longer acquisition (48). Both platforms offer rich cell-state and neighborhood information, but limited acquisition area, low throughput, instrument cost, destructive tissue measurement, and specialized analysis restrict routine deployment. At present, they are best positioned for discovery and focused translational validation rather than population-scale testing.

Spatial transcriptomics adds gene-expression state to tissue location, but platform classes should not be conflated. Standard Visium uses 55 μm capture spots that usually contain multiple cells, whereas Visium HD uses a continuous array of 2 × 2 μm barcoded squares that are commonly aggregated into larger analytical bins (49, 50). Recent TNBC studies illustrate biological applications of spatial transcriptomic profiling (51, 52). Imaging-based systems such as Xenium, MERSCOPE, and CosMx localize targeted transcripts at subcellular coordinates and can assay hundreds to several thousand genes, depending on platform and panel (33, 49). Sequencing-based methods offer broader transcriptomic depth, whereas imaging-based methods offer finer localization but targeted panels and intensive imaging. Benchmarking studies show that effective resolution is influenced by molecular diffusion, sequencing depth, transcript-detection sensitivity, and segmentation, and that performance differs across platforms and tissue preparation conditions (33, 53).

Platform choice should follow the clinical question and stage of biomarker development (**Table 2**). High-plex imaging and spatial transcriptomics are well suited to discovery; lower-plex mIF can test a defined candidate panel in larger cohorts; and H&E-based digital pathology offers the greatest scalability if an interpretable feature can be externally validated against higher-plex measurements. Recent work has extended TNBC spatial analysis to CD103+ tissue-resident memory T-cell interactions and has used artificial intelligence to infer spatial transcriptomic features from routine histology (54, 55). These advances are promising, but they remain dependent on retrospective data, reference-platform quality, and external validation.

**Table 2:** Quantitative and practical comparison of major spatial-analysis approaches relevant to TNBC.

Approach	Typical resolution and molecular depth	FFPE/scale	Reproducibility evidence	Clinical transferability and key limitations
H&E + AI	Cell- or patch-level morphology; no direct molecular plex	Routine FFPE; whole-slide; high throughput	External performance depends on scanner, staining, population, annotation, and model lock (55)	Highest scalability and lowest incremental laboratory burden; requires explainability and prospective external validation
mIHC/mIF	~0.2-0.5 μm optical resolution; commonly 4–8 markers (larger optimized panels possible)	Routine FFPE; whole-slide or large region of interest (ROI); batchable	MITRE six-site study: breast-cancer density R² ~0.75 intersite and ~0.88 intrasite; proximity R² ~0.82 (46)	Closest to clinical laboratory workflows; panel-specific validation, autofluorescence, thresholding, and analysis remain limiting
MIBI-TOF	Instrument-dependent subcellular sampling; 36-40+ proteins (12)	FFPE; selected ROIs; low throughput	Limited formal multisite concordance for complete end-to-end workflows	Strong discovery value; costly instrumentation, destructive acquisition, limited tissue area, and specialist analysis
IMC	Standard acquisition ~1 μm; commonly 30–50 proteins; high-resolution IMC (HR-IMC) below 350 nm with oversampling (48)	FFPE; selected ROIs; low throughput	Cross-study biological replication exists, but standardized multisite assay concordance remains limited	Discovery and focused validation; slow acquisition, restricted area, tissue ablation, and infrastructure cost
Sequencing-based ST	Visium: 55 μm spots; Visium HD: 2 × 2 μm capture squares, often analyzed in larger bins; transcriptome-wide (50)	Fresh frozen or FFPE depending assay; millimeter-scale capture areas	Effective resolution and sensitivity vary with diffusion, sequencing depth, tissue, and analysis (53)	Broad molecular depth; multicellular mixing or binning, batch effects, cost, and deconvolution complicate clinical use
Imaging-based ST	Subcellular transcript coordinates; targeted panels from hundreds to several thousand genes	FFPE-compatible; platform-specific field of view (FOV) and long imaging workflows	Head-to-head FFPE benchmarking shows differences in sensitivity, specificity, segmentation, and workflow (33)	High-resolution cell-state mapping; targeted panels, segmentation error, platform drift, and cost limit routine adoption

## 5 Spatial biomarkers for therapeutic prediction: evidence spanning levels 2-3

In the neoadjuvant setting, stromal TILs already provide prognostic and response-associated information (22, 29). Phase III ICI trials establish the treatment context (56), while spatial features may offer additional refinement. Intratumoral CD8+ cells, active tumor-immune interfaces, and permissive immune neighborhoods are biologically plausible correlates of response, but the supporting evidence remains predominantly retrospective or exploratory. Dynamic profiling during therapy may reveal cytotoxic expansion, contraction of inhibitory myeloid compartments, or persistence of exclusion barriers (57, 58). Such signals should be evaluated using pre-specified sampling times and endpoints rather than selected *post hoc*.

For ICIs, spatial analysis may explain why some PD-L1-positive tumors fail to respond and why some PD-L1-low tumors may still respond (3, 59). Candidate favorable features include tumor-contacting cytotoxic T cells, dendritic-cell-rich interfaces, mature TLS, and interferon-associated programs; candidate resistance features include macrophage- and Treg-rich niches, fibroblast-mediated exclusion, hypoxic stroma, and antigen-presentation loss (24, 25). Multi-checkpoint co-expression involving PD-1/PD-L1, TIM-3, LAG-3, TIGIT, or CTLA-4 may identify dysfunctional states (23, 25), but spatial co-expression is not yet a validated basis for selecting checkpoint combinations.

The same framework can generate hypotheses for rational combinations with antibody-drug conjugates, PARP inhibitors, myeloid-directed agents, or stromal interventions (25). The useful contribution of a spatial assay would be to distinguish mechanisms of failure - insufficient immune priming, impaired trafficking, physical exclusion, inhibitory myeloid signaling, T-cell dysfunction, or deficient antigen presentation - that are collapsed by a single summary score. Such mechanistic stratification should initially be used to design and enrich clinical trials, not to prescribe unvalidated combinations.

Longitudinal analysis may be a practical near-term research application because neoadjuvant treatment can generate baseline, early on-treatment, and surgical specimens. In NeoTRIP, baseline and on-treatment IMC features jointly discriminated response better than either time point alone (13). Nevertheless, paired-tissue studies are vulnerable to sampling mismatch, treatment-induced changes in tumor cellularity, and informative missingness when residual tumor is absent or scarce. Protocols therefore need anatomically matched sampling and analytical plans defined before outcome analysis.

Pathological complete response (pCR) should be treated as a biologically meaningful post-treatment state in which a residual-tumor spatial measurement is structurally undefined, not as an ordinary missing value to be imputed. A robust analysis can use a two-component strategy with separately defined estimands: first, the between-arm difference in pCR probability in the full randomized cohort, with treatment conditions and intercurrent-event strategies pre-specified; second, the between-arm difference in change in a continuous spatial score from baseline to a fixed early on-treatment biopsy collected before tumor disappearance is common. Surgical spatial features should be analyzed conditionally among patients with residual disease and reported together with the pCR proportion and residual cancer burden; a joint or hierarchical model can combine these components if a single trial endpoint is required. Repeated continuous measures can be analyzed with mixed-effects models including patient, site, and batch terms. For genuinely missing samples among patients who should have measurable tissue, multiple imputation or inverse-probability weighting should use baseline burden, treatment arm, early response, and the recorded reason for missingness, with pattern-mixture or delta-adjusted sensitivity analyses for data missing not at random (60). Design adaptations include mandated baseline and fixed early on-treatment biopsies, image-guided anatomically matched multiregion sampling, minimum tumor-area criteria, central pathology quality control, pre-specified tissue-adequacy and no-call rules, and complete recording of why each specimen is absent or nonevaluable.

Treatment de-escalation is an attractive but high-risk application. No spatial assay currently has the analytical validation, prospective evidence, or demonstrated clinical utility required to independently support treatment escalation or de-escalation in TNBC. In the near term, spatial biomarkers are more appropriately used for risk refinement, mechanistic trial stratification, and the selection of prospective endpoints. Any proposed clinical classifier should demonstrate incremental value beyond stage, residual cancer burden, stromal TILs, and PD-L1.

## 6 Implementation barriers and the roadmap to clinical translation (overarching across levels 1-3)

Several barriers must be overcome before spatial biomarkers can enter practice. Most TNBC studies remain retrospective, include modest sample sizes, and combine heterogeneous treatments, specimen types, and sampling schedules. Biomarkers identified in neoadjuvant cohorts may not transfer to metastatic disease, and features derived from ICI-treated tumors may not apply to chemotherapy-only populations. Without prospectively registered endpoints, it is difficult to determine whether a spatial feature improves prediction beyond simpler clinicopathological variables or merely correlates with them.

Pre-analytical and analytical standardization are equally important. Ischemia and fixation, section thickness, antibody clone or probe design, staining batch, image normalization, tumor-region annotation, cell segmentation, neighborhood radius, distance metric, and threshold selection can each alter a spatial score. Reproducibility should be evaluated at the level of the complete locked workflow, including local image acquisition and analysis, rather than only at a centralized analytical site. Current SITC image-analysis recommendations and the STORMI consensus checklist provide practical reporting and quality-control scaffolds (37, 38), but they do not establish a universal biological cutoff. Reports should provide continuous distributions, pre-specified cutoffs, missing-data rules, uncertainty estimates, and sensitivity analyses across plausible segmentation and threshold choices.

Clinical translation should follow the three-level maturity framework introduced at the outset (**Table 1**): standardized stromal TIL scoring is the established Level 1 measure, while compartment-aware TIL extensions, CD8+ T-cell-tumor proximity, TLS maturity, and limited-panel spatial checkpoint states are Level 2 candidates that remain promising but not standardized; high-dimensional multi-checkpoint and neighborhood signatures remain discovery-stage Level 3 features. The implementation barriers described in this section operate across all three levels and therefore do not constitute Level 4. A clinically useful classifier should contain a small number of interpretable dimensions, demonstrate multisite concordance and external validity, and show that it changes a clinically meaningful decision or outcome. Black-box performance on a held-out retrospective set is not sufficient.

## 7 Conclusion

Therapeutic response in TNBC reflects immune-cell abundance, functional state, tissue position, and temporal evolution. Spatial biomarkers can describe cytotoxic access, immune exclusion, suppressive myeloid and Treg niches, TLS organization, and checkpoint co-expression, but the evidence base remains dominated by retrospective and exploratory studies. Technology choice involves explicit trade-offs among resolution, molecular depth, tissue coverage, reproducibility, cost, and clinical scalability (**Table 2**). The field should prioritize a small number of interpretable candidates, lock their biological and computational definitions, test them across sites and platforms, and evaluate them prospectively against established clinicopathological models. Until those steps are completed, spatial biomarkers should refine biological understanding and trial design rather than independently determine treatment (**Table 1**).
